# Transcriptional profiling *of Pseudomonas aeruginosa* mature single- and dual-species biofilms in response to meropenem

**DOI:** 10.1099/mic.0.001271

**Published:** 2023-01-23

**Authors:** Farhana Alam, Jessica M. A. Blair, Rebecca A. Hall

**Affiliations:** ^1^​ Institute of Microbiology and Infection, School of Biosciences, University of Birmingham, Birmingham B15 2TT, UK; ^2^​ Institute of Microbiology and Infection, College of Medical and Dental Sciences, University of Birmingham, Birmingham B15 2TT, UK; ^3^​ Kent Fungal Group, Division of Natural Sciences, School of Biosciences, University of Kent, Canterbury CT2 7NJ, UK

**Keywords:** *Candida albicans*, *Pseudomonas aeruginosa*, meropenem, transcriptional profiling, dual-species biofilms

## Abstract

*
Pseudomonas aeruginosa* is a Gram-negative opportunistic pathogen frequently isolated from chronic infections of the cystic fibrosis lung and burn wounds, and is a major cause of antimicrobial-resistant nosocomial infections. *P. aeruginosa* is frequently co-isolated with the opportunistic fungal pathogen *Candida albicans,* with the presence of *C. albicans* in dual-species biofilms promoting tolerance to meropenem. Here, transcription profiling of mature *P. aeruginosa* single- or dual-species biofilms was carried out to understand the molecular mechanism(s) by which *C. albicans* enhances meropenem tolerance. *C. albicans* appeared to have a mild impact on the transcriptome of *P. aeruginosa* mature biofilms, with most differentially regulated genes being involved in interkingdom interactions (i.e. quorum sensing and phenazine biosynthesis). The addition of meropenem to mature single- or dual-species biofilms resulted in a significant bacterial transcriptional response, including the induction of the beta-lactamase, *ampC*, genes involved in biofilm formation. *P. aeruginosa* elicited a similar transcriptional response to meropenem in the presence of *C. albicans*, but *C. albicans* promoted the expression of additional efflux pumps, which could play roles in increasing the tolerance of *P. aeruginosa* to meropenem.

## Introduction

*Pseudomonas aeruginosa* is a Gram-negative bacterial pathogen associated with chronic infections in the cystic fibrosis (CF) lung [1] and burn wounds [2], and is a major contributor to nosocomial infections [3]. The ability of the bacterium to form biofilms is critical to the pathogenicity of *P. aeruginosa *, with biofilms being the predominant mode of growth in both the CF lung and wounds [4, 5]. Biofilms are of medical importance as they protect the microbes from the host’s innate immune system [4, 6], and significantly enhance antimicrobial resistance, with biofilms being 100–1000 times more resistant to antimicrobial therapies than their planktonic counterparts [7]. Furthermore, cells dispersed from biofilms exhibit a unique transcription profile [8], suggesting that dispersed cells represent a distinct stage that may enhance dissemination and infection progression.

*P. aeruginosa* is frequently co-isolated from sites of infection with the fungal pathogen *Candida albicans* [9]. These two microbes undergo complex interactions including direct cell–cell interactions, and through the secretion of signalling molecules and metabolites [10], the outcome of which is dependent on environmental factors. During biofilm formation, *P. aeruginosa* use the fungal hyphae as a scaffold to enhance the structure, composition and complexity of the biofilm [11, 12]. The presence of *C. albicans* in biofilms enhances the antibiotic tolerance of several important bacterial pathogens, including *P. aeruginosa* [13–15]. Enhanced antimicrobial resistance is hypothesized to result from increased extracellular matrix production in dual-species biofilms, as a result of the fungus contributing cell wall carbohydrates such as glucans and mannans to the matrix [13–16]. These fungal polysaccharides are thought to provide protection against antimicrobial agents through either limiting the diffusion of the antimicrobial through the biofilm (i.e. vancomycin) [15] or binding and sequestering the drugs (i.e. azoles) [17, 18].

Meropenem is a carbapenem antibiotic that is frequently used to treat chronic *P. aeruginosa* infections. Previously we have shown that in dual-species biofilms *C. albicans* increases the tolerance of *P. aeruginosa* to meropenem, a process dependent on fungal mannan [13]. To provide more detail on the molecular mechanism(s) underlying this phenomenon, we analysed the transcriptional response of mature *P. aeruginosa* biofilms, and dual-species biofilms in the absence and presence of meropenem. The transcriptional profile identifies key *P. aeruginosa* genes and biological processes required for resistance to meropenem. Similar genes and processes were unregulated by *P. aeruginosa* in dual-species biofilms, indicating that the presence of *C. albicans* in mature biofilms does not perturb the *P. aeruginosa* transcriptional response to meropenem.

## Methods

### Strains and media

*C. albicans* SC5314 was grown and maintained on yeast peptone dextrose (YPD; Sigma) media, while *P. aeruginosa* PAO1 (ATCC15692) was grown and maintained on Luria-Bertani (LB) medium. All biofilm assays were performed in Muller–Hinton broth (MHB).

### Biofilm assay

Biofilm assays were based on previously described methodology [13], but scaled up to six-well plates. In brief, overnight cultures of *C. albicans* and *P. aeruginosa* were washed in phosphate-buffered saline (PBS), and *C. albicans* resuspended at 1×10^6^ cells ml^−1^ and *P. aeruginosa* to an OD_600_ of 0.2 [~2×10^8^ colony-forming units (c.f.u.) ml^−1^] in Mueller–Hinton broth. Each well contained 3 ml *C*. *albicans* and 300 µl of *P. aeruginosa* in a total of 6 ml. Plates were incubated at 37 °C for 2 h to allow cells to adhere, at which point the media were replaced with fresh sterile media, and plates were incubated statically at 37 °C for 24 h. Cells not part of the biofilm were removed, media were replaced with fresh MHB containing 0 or 5 µg ml^−1^ meropenem, and plates were incubated for 4 h. Media were replaced with 2 ml PBS containing 50 µg ml^−1^ DNase I and plates were incubated at 37 °C for 1 h to degrade the extracellular matrix. Biofilms were detached from the plate by scraping, serially diluted and plated onto selective agar (YPD agar supplemented with 100 µg ml^−1^ tetracycline to determine viable *C. albicans* c.f.u. and cetrimide agar to determine viable *P. aeruginosa* c.f.u.).

### Preparation of samples for RNA extraction

Biofilms were formed as described above and triplicate biofilms were pooled, 50 µl serially diluted and plated on cetrimide agar or YPD supplemented with 100 µg ml^−1^ tetracycline to check for contamination. Remaining biofilm cells were centrifuged at 3500 r.p.m at 4 °C for 5 min and pellets snap frozen in liquid nitrogen. Four biological replicates were shipped to GeneWiz, UK, for RNA extraction sequencing and basic bioinformatic analysis.

### RNA extraction and sequencing

RNA was extracted from the biofilms by GeneWiz using the Qiagen RNeasy Plus mini kit. Library preparation was done in the following stages: (a) ribosomal RNA depletion; (b) RNA fragmentation and random priming; (c) first and second strand cDNA synthesis; (d) end repair, 5′ phosphorylation and dA-tailing; (e) adapter ligation, PCR enrichment and sequencing. Paired-end sequencing was performed using Illumina HiSeq 4000 (2×150 bp configuration, single index per lane).

### Bioinformatic analysis

The sequence quality of each sample was evaluated by determining the number of reads, the yield (Mbases), the mean quality score and the percentage of reads over 30 bases in length. FastQC software was used to determine per-base-sequence quality and per-sequence GC content. Sequence reads were trimmed to remove adapter sequences and nucleotides with poor quality using Trimmomatic v.0.36. The trimmed reads were mapped to the *P. aeruginosa* reference genome, available on ENSEMBL, using the STAR aligner v.2.5.2b. For dual-species biofilms, samples were treated the same as single-species biofilms. Reads were mapped to the PAO1 genome, and non-mapped reads were discarded and later aligned to the *C. albicans* reference genome. Unique gene hit counts were calculated using featureCounts from the Subread package v.1.5.2. Only unique reads that fell within exonic regions were counted (for unique genes, the number of hits per read was set to 10 as default, with reads that mapped to <10 distinct places assigned to one place by the EM algorithm, and reads that mapped to >10 distinct places being discarded). For the analysis, only read counts for genes in the *P. aeruginosa* genome were used and the TPM values were calculated as follows: each read count was divided by the length of each gene in kb to generate reads per kb (RPK), then all the RPK values in the sample were counted and divided by 1 000 000 to generate the scaling factor, and finally the RPK values were divided by the scaling factor to generate the TPM value. Differential gene expression analysis was performed using DESeq2 and the comparisons listed in Table 1. Principal component analysis (PCA) was performed to reveal the similarities within and between groups, with PCA plots included in the output (Fig. S1, available with the online version of this article). As expected, dual-species biofilms exhibited greater biological variation than single-species biofilms (Fig. S1), which likely reflects the heterogeneity of the biofilm structure. Meropenem treatment had the greatest effect on the bacteria transcriptome, with samples clustering into distinct groups in the PCA plots, while *C. albicans* had a reduced impact on the *P. aeruginosa* transcriptome, resulting in great spread of the data. DESeq2 output files for each comparison, and files containing summary raw and normalized reads and TPM values for each gene are available at the Gene Expression Omnibus (GEO) database (https://www.ncbi.nlm.nih.gov/geo/), under accession number GSE167137.

**Table 1.** Differential expression analysis carried out by DESeq2

Comparison	Abbreviation	Output file
Untreated *P. aeruginosa* mono-species biofilm vs *P. aeruginosa* mono-species treated with 5 µg ml^−1^ meropenem	PA_0M vs PA_5M	P_aeruginosa_expression_ PA_0M_vs_PA_5M_.xlsx
Untreated *P. aeruginosa* mono-species biofilm vs dual-species biofilms	PA_0M vs PACA_0M	P_aeruginosa_expression_ PA_0M_vs_PACA_0M.xlsx
Untreated *P. aeruginosa* mono-species biofilms vs dual-species biofilms treated with 5 µg ml^−1^ meropenem	PACA_0M vs PACA 5M	P_aeruginosa_expression_ PACA_0M_vs_PACA_5M.xlsx

### Enrichment analysis

For *P. aeruginosa* transcriptomic analysis, differential expression of genes between conditions was considered significant if the adjusted *P*-value (Padj) was ≤0.05. Gene ontology (GO) analysis was performed using KOBAS 3.0 software [Kyoto Encyclopaedia of Genes and Genomes (KEGG) Orthology Based Annotation System] [19].

### Statistical analysis

Biofilm data were analysed in GraphPad Prism (version 9.1.0) using two-way analysis of variance (ANOVA) and Holm–Sidak’s multiple comparisons test.

## Results

### *C. albicans* enhances the tolerance of *P. aeruginosa* biofilms to meropenem even at early timepoints

**Fig. 1.**
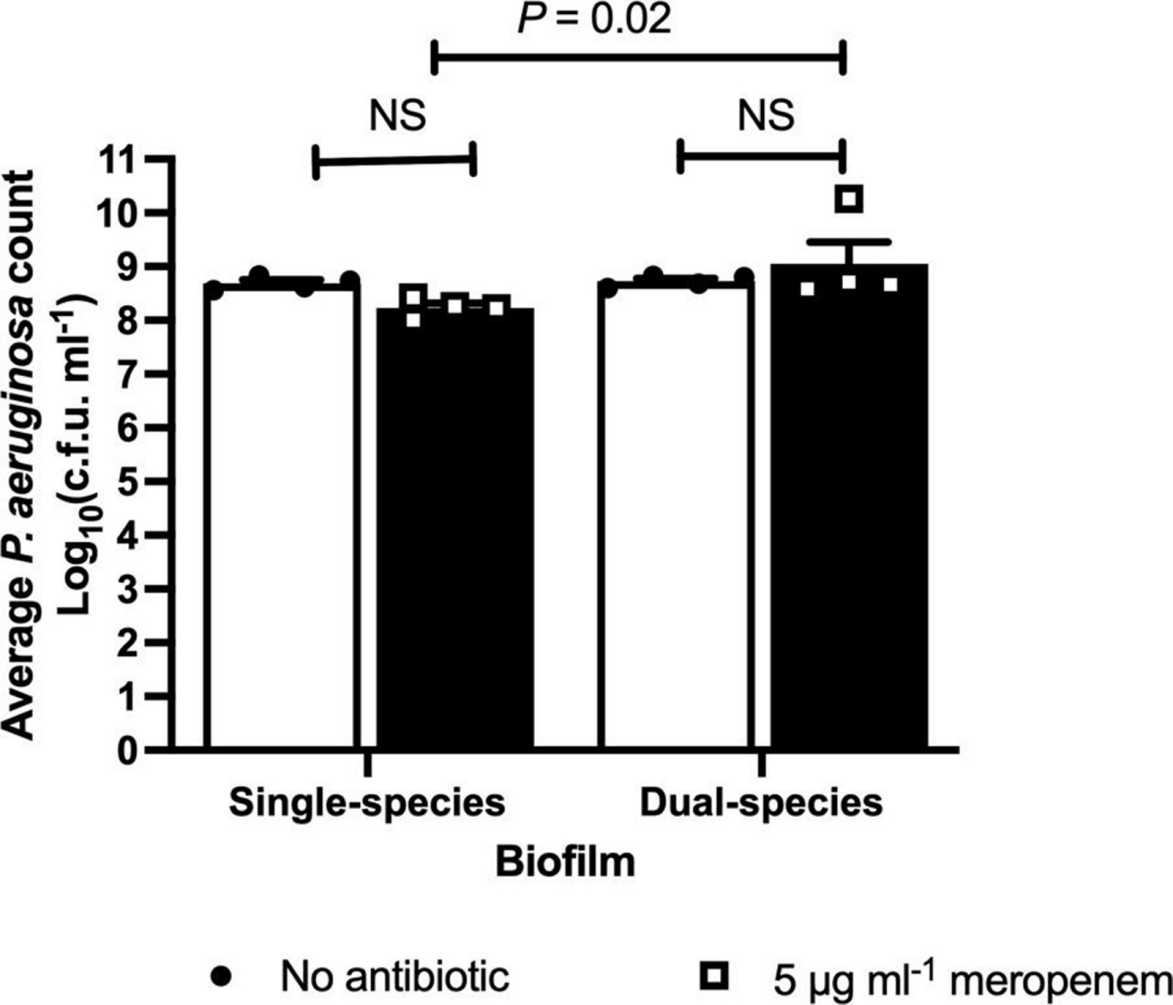
*C. albicans* enhances the tolerance of *P. aeruginosa* to meropenem after short exposures. Preformed 24 hr biofilms were incubated in Mueller Hinton broth containing 0 or 5 μg/mL meropenem, for 4 hr and viable *P. aeruginosa* counts quantified. Data are the mean ± the SEM from 4 biological replicates. Data were analysed using 2-way ANOVA and Holm-Sidak’s multiple comparisons test (ns not significant; *P < 0.05).

Previously we have observed that when *P. aeruginosa* is grown in a dual-species biofilm with the fungal pathogen *C. albicans*, the tolerance of *P. aeruginosa* to meropenem is increased [13]. To understand the molecular mechanism(s) behind this increased tolerance, transcriptional profiling was performed. To avoid the transcriptional profile focusing on genes related to cell death, we analysed the transcriptome after the antibiotic had been added to mature biofilms for 4 h. Plating of mono- and dual-species biofilms in the absence and presence of meropenem confirmed that the majority of the cell population was viable at this time point. Furthermore, the tolerance of *P. aeruginosa* to meropenem in the presence of *C. albicans* was still enhanced even at this early timepoint (Fig. 1).

### Meropenem enhances *ampC* expression in *P. aeruginosa* single-species biofilms

**Fig. 2.**
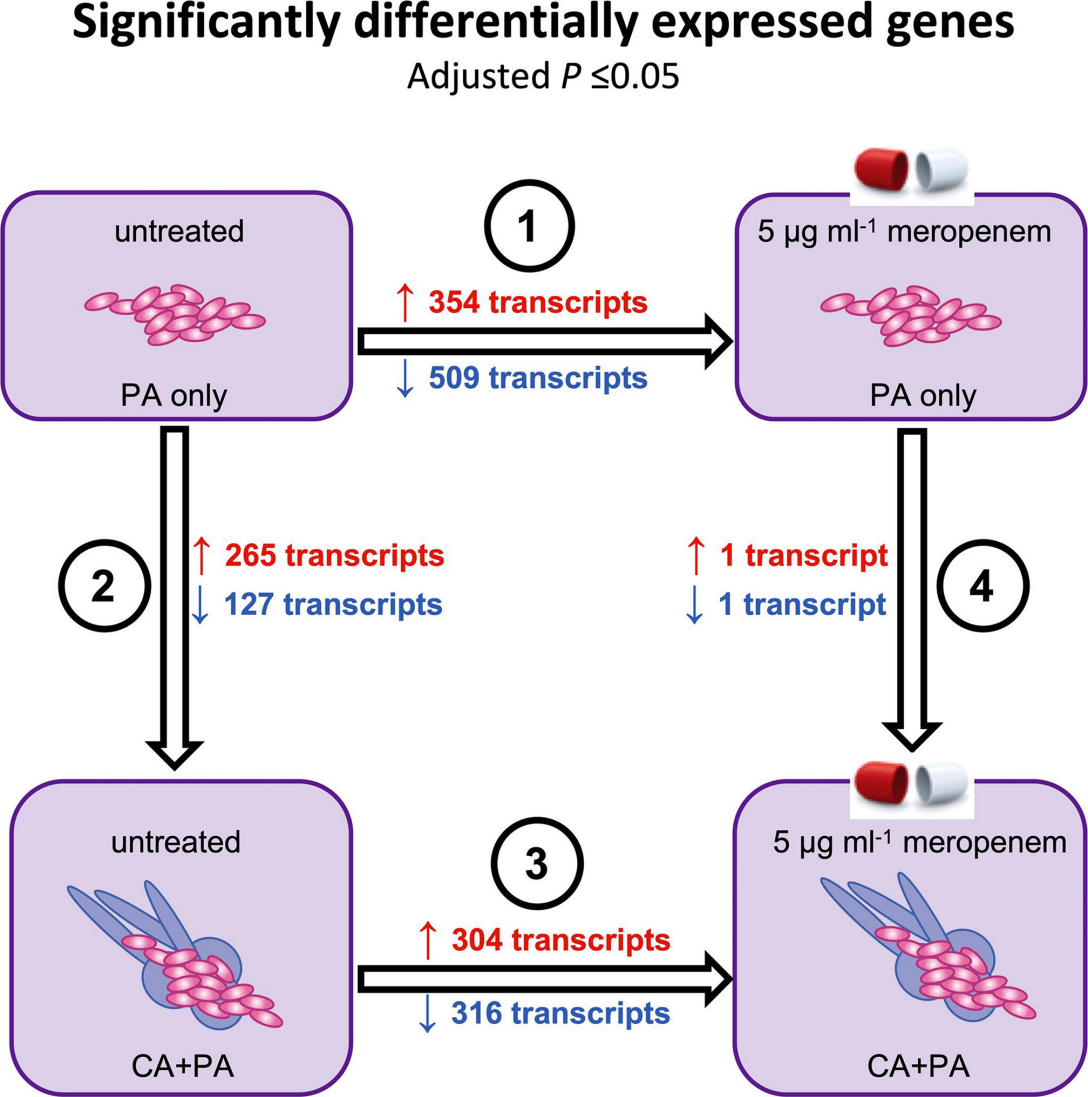
Summary of transcriptional responses of *P. aeruginosa* biofilm cells to C. albicans and to meropenem treatment. Numbers of significantly differentially expressed *P. aeruginosa* transcripts (Padj ≤ 0.05) in 1) *P. aeruginosa* single-species biofilms treated with 5 μg ml^-1^ meropenem; 2) dual species untreated biofilms; 3) dual species biofilms treated with 5 μg ml^-1^ meropenem; 4) single vs dual species biofilms treated with 5 μg ml^-1^ meropenem. PA: *P. aeruginosa *; CA: *C. albicans*. Red: numbers of significantly upregulated transcripts; Blue: numbers of significantly downregulated transcripts.

**Fig. 3.**
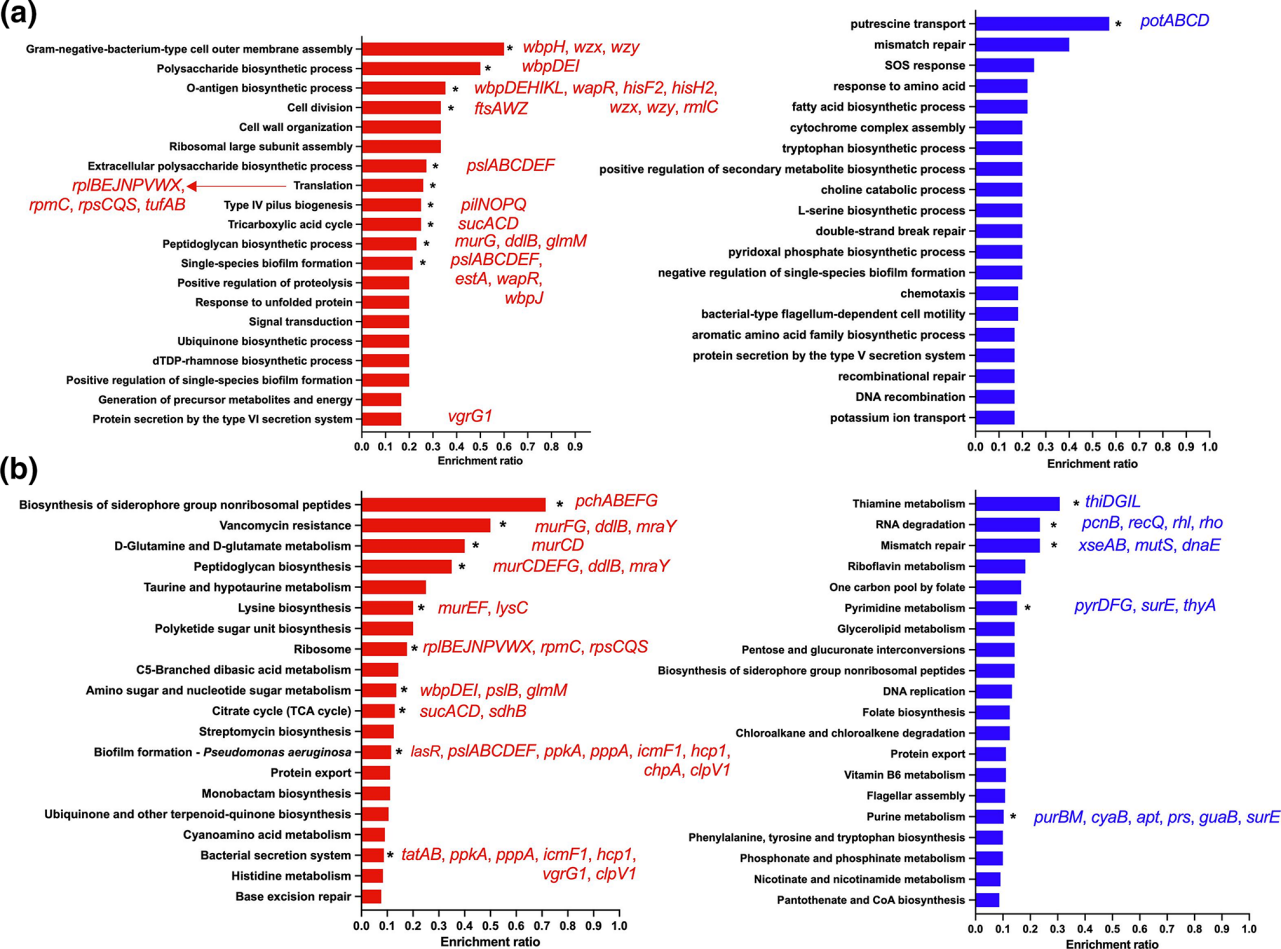
Meropenem treatment increases the expression of genes required for biofilm formation and iron homeostasis. (a) GO term enrichment analysis and (b) KEGG analysis of genes significantly differentially regulated in *P. aeruginosa* single-species biofilms by meropenem. Bars in red represent pathways/processes that were significantly upregulated, while bars in blue represent pathways/processes that were significantly downregulated. Data were analysed using Fisher’s exact test and the Benjamini–Hochberg method for FDR correction (*, FDR≤0.05).

Addition of meropenem to *P. aeruginosa* mono-species biofilms resulted in the significant upregulation of 354 genes, while 509 genes were downregulated (Figs 2 and S2a, Table 2). Of the significantly differentially regulated genes, 45 (159/354) and 43 % (217/509) encoded hypothetical proteins. As expected, the most significantly upregulated gene in response to meropenem treatment was *ampC* (log_2_ fold change=7.75, Padj=1.9510^−42^), which encodes a beta-lactamase, important for carbapenem resistance. GO term enrichment analysis of the differentially regulated genes confirmed that meropenem resulted in the significant upregulation of genes involved in the maintenance of the bacterial cell membrane, cell wall, biofilm formation and extracellular matrix production, siderophore production, type IV pilus formation and type VI secretion system, while genes involved in putrescine transport were significantly downregulated (Fig. 3a). KEGG pathway analysis identified the over-representation of genes involved in siderophore biosynthesis, vancomycin resistance, amino acid metabolism, biofilm formation and bacterial secretion in genes that were significantly upregulated and pathways associated with thiamine metabolism, mismatch repair, pyrimidine metabolism and purine metabolism were significantly downregulated (Fig. 3b).

**Table 2.** Top 20 differentially regulated genes in *P. aeruginosa* untreated single biofilms vs single-species biofilms treated with 5 µg ml^−1^ meropenem

	Gene ID	log_2_ fold change	*P*-value	Padj
**Upregulated**	*ampC*	5.745634677	3.54E-46	1.95E-42
	*PA4111*	3.141752798	2.08E-25	1.27E-22
	*hcp1*	2.299594301	9.57E-20	2.64E-17
	*PA0126*	2.010278388	4.77E-15	6.75E-13
	*clpV1*	1.832477469	5.89E-09	3.65E-07
	*PA4280.2*	1.825681731	0.0007200374797	0.008213485384
	*PA0084*	1.81927681	9.59E-07	3.60E-05
	*PA0083*	1.812545239	2.64E-06	8.69E-05
	*PA4690.2*	1.811643426	0.0006939310453	0.00798165271
	*PA5369.2*	1.811071763	0.0007048977579	0.008057433792
	*PA0668.4*	1.770903731	0.0005529877155	0.006709989401
	*PA0070*	1.759966355	2.86E-06	9.28E-05
	*PA0277*	1.706435238	2.22E-11	2.04E-09
	*vgrG1*	1.692796239	4.23E-08	2.12E-06
	*PA0466*	1.68245735	3.45E-06	0.0001075909918
	*PA0089*	1.645610422	8.37E-08	3.89E-06
	*PA0050*	1.596317219	3.73E-05	0.0007623159776
	*rplX*	1.577791178	0.003955658099	0.03020634629
	*rpsQ*	1.514302023	0.003135531829	0.0252719288
	*rplN*	1.482472974	0.006174262969	0.04190492586
**Downregulated**	*PA0627*	−2.563807744	7.94E-27	6.26E-24
	*PA0629*	−2.554150934	7.27E-33	1.34E-29
	*PA0641*	−2.55319332	1.46E-27	1.34E-24
	*PA0628*	−2.534582315	1.07E-31	1.47E-28
	*PA0638*	−2.46361566	3.36E-33	9.27E-30
	*PA0636*	−2.444427878	2.44E-22	1.04E-19
	*PA0635*	−2.415846681	1.41E-22	6.50E-20
	*gfnR*	−2.353411699	6.68E-14	8.58E-12
	*PA0630*	−2.310960008	2.67E-21	8.68E-19
	*PA0639*	−2.28619203	5.70E-25	2.86E-22
	*PA3638*	−2.285364587	4.83E-11	4.17E-09
	*PA0634*	−2.272274644	9.09E-19	2.09E-16
	*PA1168*	−2.27006418	2.26E-06	7.65E-05
	*PA0637*	−2.231450235	9.63E-22	3.80E-19
	*PA0616*	−2.222013132	1.13E-25	7.79E-23
	*PA0625*	−2.213888097	1.84E-21	6.78E-19
	*ptrB*	−2.203953211	6.10E-17	1.05E-14
	*PA0631*	−2.195277667	2.16E-20	6.63E-18
	*PA0613*	−2.187796631	4.07E-18	8.32E-16
	*PA0618*	−2.143020354	3.73E-18	7.91E-16

### The presence of *C. albicans* in dual-species biofilms results in mild changes in the *P. aeruginosa* transcriptome

**Fig. 4.**
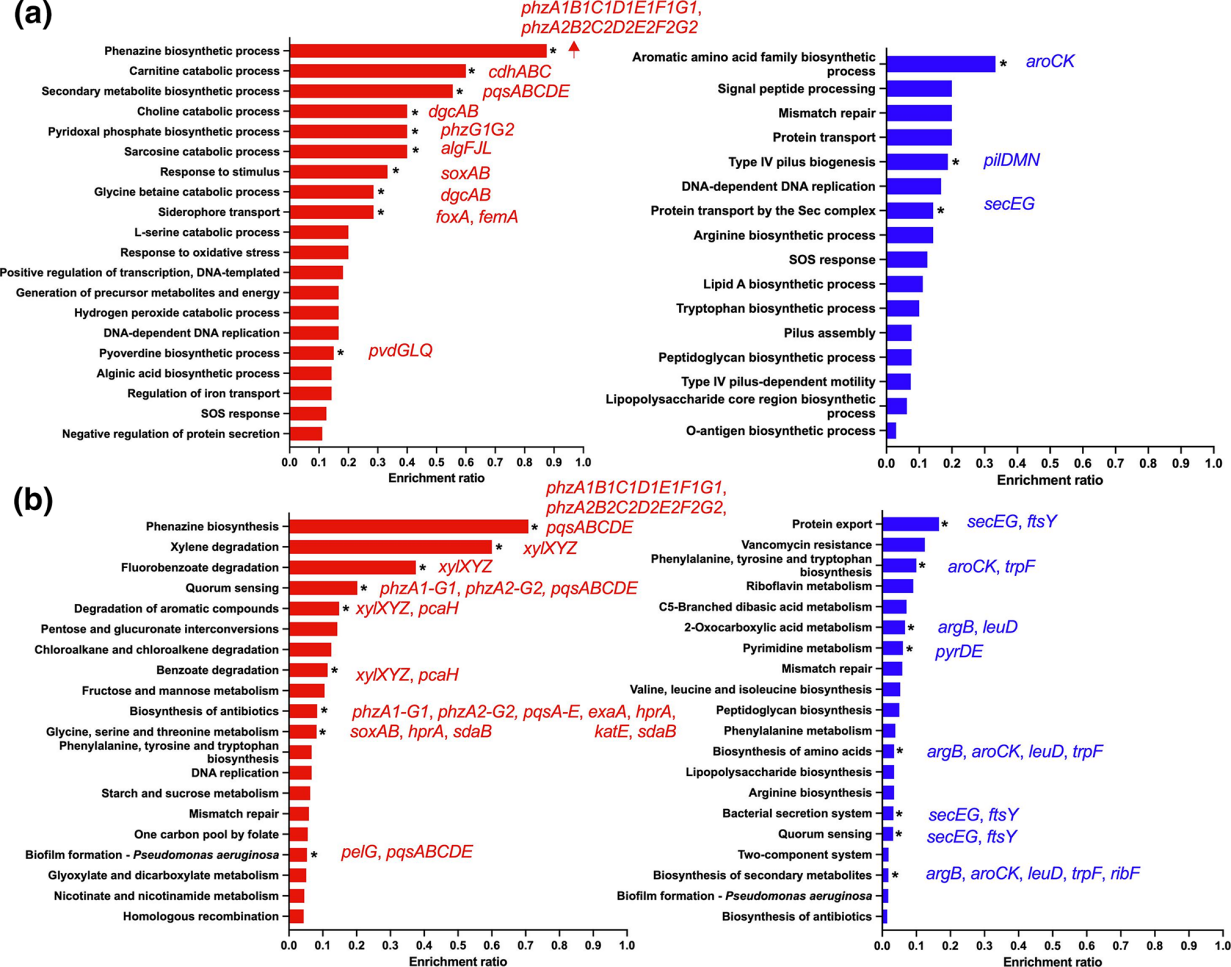
*
P. aeruginosa* upregulates genes that provide a competitive fitness advantage during growth in dual-species biofilms. (a) GO term enrichment analysis and (b) KEGG analysis of *P. aeruginosa* genes significantly differentially regulated in dual-species biofilms. Bars in red represent pathways/processes that were significantly upregulated, while bars in blue represent pathways/processes that were significantly downregulated. Data were analysed using Fisher’s exact test and the Benjamini–Hochberg method for FDR correction (*, FDR≤0.05).

To determine how the presence of *C. albicans* affected *P. aeruginosa *, we compared the transcriptional profile of *P. aeruginosa* single-species biofilms to dual-species biofilms. The presence of *C. albicans* resulted in the differential regulation of 392 genes, with 265 genes being significantly upregulated and 127 genes being significantly downregulated (Figs 2 and S2b, Table 3). Although significant, we note that the log_2_ values for the majority of these genes were <1, suggesting that *C. albicans* has a mild impact on the transcription profile of *P. aeruginosa *. The most significantly differentially regulated genes were PA4097 (a lipolytic protein) and PA5384 (an alcohol dehydrogenase), which were both twofold upregulated in the presence of *C. albicans*. GO term enrichment analysis (Fig. 4a) and KEGG pathway analysis (Fig. 4b) of the 392 differentially regulated genes identified that phenazine biosynthesis, *Pseudomonas* quinolone signal (PQS) production, pyridocal phosphate biosynthesis, and siderophore transport and pyoverdine biosynthesis were significantly upregulated, while genes involved in type IV pilus biogenesis, the general secretory (Sec) pathway and amino acid biosynthesis were significantly downregulated. Therefore, *P. aeruginosa* appears to upregulate processes that might provide the bacterium with a competitive advantage when growing in the presence of *C. albicans*.

**Table 3.** Top 20 differentially regulated *P. aeruginosa* genes in dual-species verses single-species biofilms

	Gene ID	log_2_ fold change	*P*-value	Padj
**Upregulated**	*PA5384*	1.136608573	4.28E-06	0.0002779376198
	*PA4097*	1.000660434	3.17E-05	0.001214321657
	*cdhB*	0.9811955186	5.21E-06	0.0003198899691
	*PA2161*	0.9810583138	0.0003525619038	0.008808371695
	*cdhC*	0.936864827	9.18E-07	7.92E-05
	*cdhA*	0.9176339588	1.17E-09	4.57E-07
	*ssrS*	0.9064141369	8.59E-13	6.76E-10
	*PA0698*	0.9014266847	1.42E-05	0.0006654679167
	*PA2349*	0.8950640392	2.59E-05	0.001067775173
	*PA2181*	0.8814108328	9.08E-08	1.36E-05
	*PA2090*	0.8800287264	1.03E-07	1.50E-05
	*PA2324*	0.867636224	1.23E-05	0.0005976988392
	*PA2213*	0.8532908266	7.35E-16	1.35E-12
	*PA2180*	0.8294884121	1.24E-09	4.57E-07
	*PA4088*	0.8176661995	7.06E-09	1.77E-06
	*czcC*	0.8158365062	9.15E-08	1.36E-05
	*phzC2*	0.7984412293	3.58E-07	4.21E-05
	*phzC1*	0.788317377	3.04E-05	0.00117677973
	*PA4098*	0.7866314328	0.0005949449343	0.01341483781
	*phzA1*	0.7731714023	4.59E-06	0.0002943805348
**Downregulated**	*PA3572*	−0.9273548369	0.003059228691	0.0450911657
	*argB*	−0.8972467441	3.86E-09	1.02E-06
	*rnpB*	−0.8480655754	3.66E-08	5.77E-06
	*PA1746*	−0.7808094482	2.35E-05	0.0009898437811
	*PA4351*	−0.7261070956	2.78E-05	0.00110592551
	*PA5303*	−0.6697670409	3.36E-13	3.09E-10
	*ftsY*	−0.5672143027	1.04E-08	2.29E-06
	*PA4276.1*	−0.5616231924	1.53E-05	0.0006970521393
	*PA4690.3*	−0.5391253382	0.002180107776	0.03467439429
	*PA1137*	−0.5377222343	1.98E-11	9.95E-09
	*PA3133.3*	−0.5289323628	0.0003527179099	0.008808371695
	*ssrA*	−0.5226447106	3.43E-06	0.0002369715977
	*PA3573*	−0.5188361793	6.23E-05	0.002219824419
	*PA4421*	−0.5151637325	1.87E-11	9.95E-09
	*PA1796.1*	−0.4988013205	0.001465215919	0.02591835467
	*PA4637*	−0.4958503362	0.001596304616	0.02744549898
	*PA1510*	−0.4875966167	3.42E-08	5.55E-06
	*PA3951*	−0.4870639148	9.51E-09	2.25E-06
	*PA2943*	−0.4748718073	1.76E-06	0.0001407992501
	*PA3277*	−0.4616098795	0.0007070580094	0.01530295354

To identify whether the presence of the fungus was inducing a similar transcriptional response to the drug treatment we compared DEGs identified in *P. aeruginosa* single-species biofilms treated with meropenem to the list of DEGs identified in untreated dual-species biofilms. Seven genes (PA10907, PA4830, PA4518, PA1209, *rimK*, PA0881 and PA3758) were upregulated, while five genes (*pyrD*, *aroC*, PA4633, PA3526 and PA4637) were downregulated under both conditions. However, as the majority of the genes encode hypothetical proteins, the impact of these transcriptional responses is unknown.

### Impact of *C. albicans* on the *P. aeruginosa* transcriptional response to meropenem

**Fig. 5.**
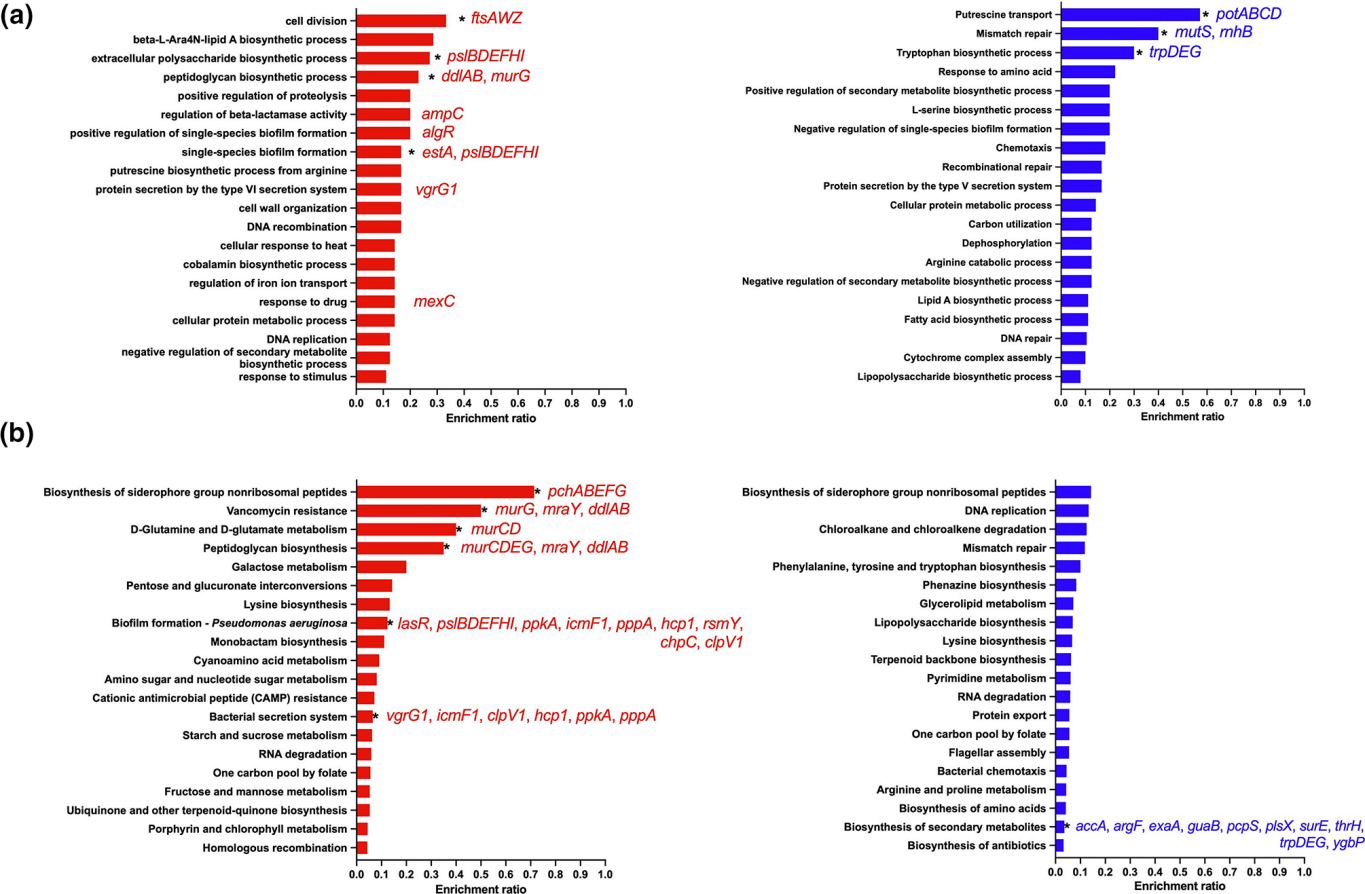
Dual-species biofilms do not perturb the *P. aeruginosa* transcriptional response to meropenem. (a) GO term enrichment analysis and (b) KEGG analysis of *P. aeruginosa* genes significantly differentially regulated in dual-species biofilms in response to meropenem treatment. Bars in red represent pathways/processes that were significantly upregulated, while bars in blue represent pathways/processes that were significantly downregulated. Data were analysed using Fisher’s exact test and the Benjamini–Hochberg method for FDR correction (*, FDR≤0.05).

To determine whether the presence of *C. albicans* affected the response of *P. aeruginosa* to meropenem, we compared the transcriptional profile of untreated dual-species biofilms to that of meropenem-treated dual-species biofilms. In response to meropenem, a total of 620 genes were differentially regulated, with 304 genes being significantly upregulated and 316 being significantly downregulated (Figs 2 and S2c, Table 4). As was the case for the single-species biofilms, *ampC* was the most significantly upregulated gene (log_2_=5.52, Padj=1.9510^−47^). GO term enrichment analysis (Fig. 5a) and KEGG pathway analysis (Figs 4 and 5b) confirmed that as with single-species biofilms, treatment of *P. aeruginosa* dual-species biofilms with meropenem resulted in the upregulation of genes involved in cell division, peptidoglycan biosynthesis, biofilm formation, extracellular matrix production, siderophore biosynthesis and type VI secretion. Likewise, the processes that were significantly downregulated in single-species biofilms in response to meropenem (i.e. putrescine transport and mismatch repair) were shared with dual-species biofilms (Fig. 5).

**Table 4.** Top 20 differentially regulated *P. aeruginosa* genes in dual-species biofilms treated with 5 µg ml^−1^ meropenem vs untreated dual-species biofilms

	Gene ID	log_2_ fold change	*P*-value	Padj
**Upregulated**	*ampC*	5.515387278	7.06E-51	1.95E-47
	*PA4280.5*	3.700797106	7.82E-06	0.0002259666295
	*PA0668.1*	3.67825942	8.05E-06	0.0002315796869
	*PA4690.5*	3.49778215	2.92E-05	0.0007129969543
	*PA5369.5*	3.485074044	3.01E-05	0.0007281076683
	*PA4111*	2.963866877	8.30E-18	1.99E-15
	*PA4690.2*	2.605990986	0.003138749477	0.03261549984
	*PA5369.2*	2.604799667	0.003142239391	0.03261549984
	*PA4280.2*	2.596702641	0.003658501903	0.03633497754
	*PA0668.4*	2.58797494	0.003177734857	0.03286039678
	*hcp1*	2.353244054	1.14E-22	9.02E-20
	*PA0466*	1.908730924	1.39E-11	1.30E-09
	*pchG*	1.899939854	1.07E-08	6.09E-07
	*PA0126*	1.883859984	5.23E-13	6.57E-11
	*PA0050*	1.820434031	2.26E-06	7.78E-05
	*clpV1*	1.75537326	1.53E-11	1.37E-09
	*vgrG1*	1.730154359	3.27E-12	3.37E-10
	*PA4222*	1.708263864	2.26E-12	2.45E-10
	*PA0563*	1.691305244	1.68E-11	1.47E-09
	*PA0086*	1.677560955	4.89E-10	3.46E-08
**Downregulated**	*PA0627*	−2.524464134	1.52E-15	2.79E-13
	*gfnR*	−2.418533396	8.72E-23	8.02E-20
	*PA0641*	−2.404904976	2.04E-20	7.49E-18
	*PA0638*	−2.364308134	4.30E-21	1.98E-18
	*PA0628*	−2.298437047	1.97E-20	7.49E-18
	*PA0635*	−2.263949045	1.95E-16	3.84E-14
	*PA0629*	−2.260720569	5.93E-19	1.92E-16
	*PA0639*	−2.220599378	2.18E-21	1.21E-18
	*PA0636*	−2.211742782	4.17E-18	1.15E-15
	*PA0625*	−2.099597686	3.91E-17	8.30E-15
	*PA0616*	−2.045775386	2.09E-15	3.72E-13
	*PA3638*	−2.041401724	2.33E-18	7.14E-16
	*PA0634*	−1.982462548	1.30E-17	3.00E-15
	*PA1168*	−1.971155931	1.76E-14	2.56E-12
	*PA0631*	−1.92975403	4.75E-15	7.95E-13
	*PA0619*	−1.919212071	1.02E-13	1.31E-11
	*PA3631*	−1.891142906	4.13E-19	1.43E-16
	*PA0626*	−1.882097335	5.95E-17	1.22E-14
	*PA0630*	−1.820934828	4.77E-11	3.71E-09
	*PA0637*	−1.809932956	1.14E-14	1.75E-12

**Fig. 6.**
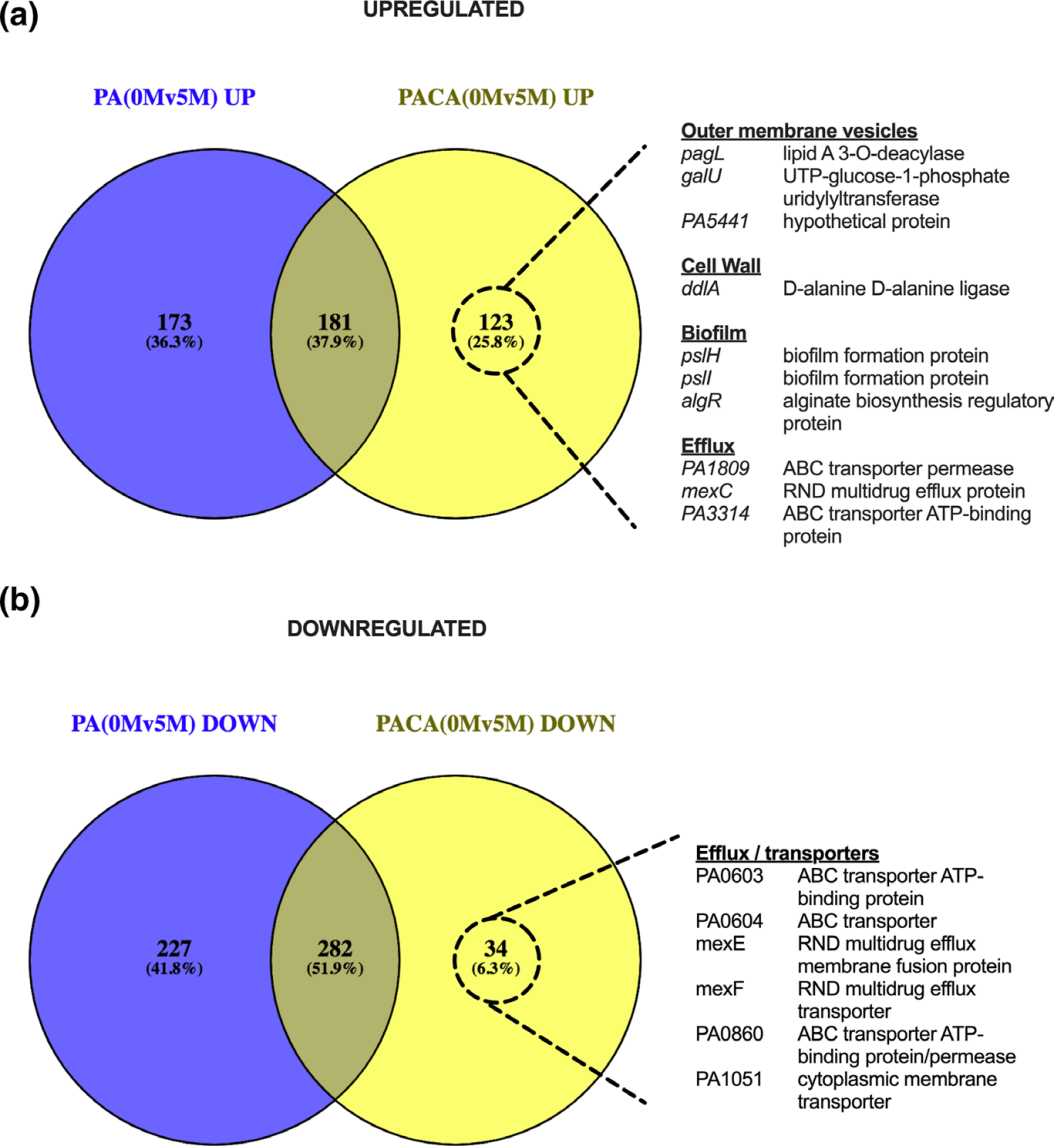
Identification of overlapping differentially expressed genes in *P. aeruginosa* mono and dual species biofilms in response to meropenem. Numbers of significantly upregulated (a) and downregulated (b) genes (Padj ≤ 0.05), in response to treatment with 5 μg/mL meropenem, are compared between single- and dual-species biofilms (PA *P. aeruginosa *; CA *C. albicans*). Genes of interest, unique to the dual-species condition, are listed on the right-hand side.

Although *C. albicans* does not affect the general processes that are differentially regulated in response to meropenem, it is possible that the underlying DEGs are different. Therefore, we compared the DEGs between single- and dual-species biofilms in response to meropenem. Of the genes that were significantly upregulated in response to meropenem, 181 (37.9 %) were upregulated in both single- and dual-species biofilms, while 173 (36.3 %) and 123 (25.8 %) were unique to single- and dual-species biofilms, respectively (Fig. 6a). Among the upregulated genes that were unique to the dual-species meropenem response, three were linked to outer-membrane vesicles (OMVs; *pagL*, *galU* and PA5441) [20], one was involved in cell wall synthesis (*ddlA*), three were involved in biofilm formation (*pslH*, *pslI* and *algR*) and three were involved in efflux (PA1809, *mexC* and PA3314). This suggests that the presence of *C. albicans* may increase the ability of *P. aeruginosa* to form robust biofilms, to secrete molecules via OMVs and upregulate the MexCD-OprJ efflux pump, which may play a role in increasing the tolerance of the bacterium to meropenem.

Of the genes that were significantly downregulated in response to meropenem, 282 (51.9 %) genes were shared in common between single- and dual-species *P. aeruginosa* biofilm cells, while 227 (41.8 %) and 34 (6.3 %) were unique to single- and dual-species biofilms, respectively (Fig. 6b). Among the few downregulated genes that were unique to the dual-species meropenem response, there were six involved in efflux or membrane transport (PA0603, PA0604, *mexE*, *mexF*, PA0860 and PA1051). This suggests that the presence of *C. albicans* may result in reduced production of the MexEF-OprN efflux pump in *P. aeruginosa* biofilm cells.

## Discussion

Biofilms are medically important, as they result in antimicrobial resistance and protect microbes from the actions of the immune system, making infection harder to treat. Furthermore, biofilms are normally mixed-species communities, with our understanding of how microbe–microbe interactions within these communities affect disease progression being limited. We have previously shown that *C. albicans* enhances the tolerance of *P. aeruginosa* to meropenem, a commonly used carbapenem for the treatment of chronic *P. aeruginosa* infections. Therefore, transcriptional analysis was carried out to understand how *C. albicans* promotes meropenem tolerance.

Mechanisms of resistance to the carbapenem class of antibiotics include decreased outer membrane permeability, beta-lactamase expression, increased efflux and target modification. In agreement with this, the addition of meropenem to mature *P. aeruginosa* single-species biofilms resulted in the significant induction of *ampC*, a beta-lactamase precursor. Differential regulation of *ampC* is linked to the natural resistance of *P. aeruginosa* to this class of antibiotic [21]. Imipenem, another carbapenem used to treat *P. aeruginosa* infections, also induces the expression of *ampC* [22], highlighting *ampC* induction as a conserved response to carbapenems. In addition to *ampC*, the outer-membrane porin, OprD, has also been associated with meropenem resistance. OprD facilitates entry of carbapenems into bacterial cells, and is therefore frequently downregulated in meropenem-resistant strains [21]. In our study, *oprD* was not differentially regulated in response to meropenem treatment. However, there is often a poor correlation between *oprD* mRNA levels and protein levels [21]. Therefore, it is possible that OprD protein levels are downregulated in our biofilms to reduce meropenem uptake.

Interestingly, treatment of *P. aeruginosa* biofilms with either meropenem or imipenem, results in the significant induction of biofilm associated genes [22]. For example, in response to meropenem extracellular polysaccharide biosynthetic genes such as *pslB*, *pslD*, *pslE* and *pslF* were significantly upregulated. The induction of these polysaccharide genes results in enhanced intracellular and cell–substrate interactions, and the expression of subsets of these genes are commonly enhanced in biofilm-forming clinical isolates. Psl is thought to enhance the tolerance of *P. aeruginosa* biofilms to a range of antimicrobial agents including colistin, tobramycin and ciprofloxacin [23].

In response to meropenem, genes involved in the type 6 secretion system (T6SS) were also significantly upregulated, including *ppkA*, *pppA*, *clpV1 icmF1*, *vgrG1* and *hcp1*. The structure of the T6SS bears similarities to the cell-puncturing needle of bacteriophage viruses; the roles of the T6SS in *P. aeruginosa* include virulence within hosts, delivery of toxins to neighbouring microbes that are competing for resources, and biofilm formation [24, 25]. Although there has been no previous research linking antibiotic treatment to upregulation of T6SS activity in *P. aeruginosa *, the T6SS has been implicated in drug resistance in *A. baumannii* and *K. pneumoniae* [26, 27]. However, in *P. aeruginosa *, the T6SS is upregulated during competition with other bacteria as a result of kin cell lysis [28]. Therefore, it is possible that the observed increase in transcription of components of the T6SS is a result of meropenem-induced cell lysis.

During growth in dual-species biofilms, *P. aeruginosa* upregulates genes required for quorum sensing, with all genes from the *pqsABCDE* operon being significantly upregulated. The *pqs* operon encodes several enzymes required for production of the *Pseudomonas* quinolone signal (PQS) [29]. *C. albicans*-dependent regulation of PQS production is complex. The fungal quorum-sensing molecule, farnesol, inhibits PQS production in planktonic interactions through modulation of PqsR-dependent transcription of the *pqs* operon [30]. However, in *lasR*-deficient *P. aeruginosa* strains, *C. albicans* restores PQS production through farnesol-induced ROS-dependent production of C4-HSL [31]. Increased C4-HSL results in the induction of pqsH and therefore restores PQS and phenazine production [31]. However, PQS is also induced under iron-limiting conditions [32], resulting in increased biosynthesis of phenazines and siderophores, which both scavenge iron from the environment.

Phenazines are redox-active molecules that affect the redox balance of cells, the uptake of metabolites and gene expression, and have also been shown to enhance *P. aeruginosa* tolerance to the antibiotic ciprofloxacin [33]. Phenazines are also able to facilitate electron transfer within biofilms [34]. Pathways associated with the biosynthesis of these molecules were the most significantly upregulated biological process in our dual-species biofilms, and in other studies highlighting their importance in interkingdom interactions.

Our transcriptional analysis identified several RND and ABC transporters to the differentially regulated in dual-species biofilms in the presence of meropenem. Little is known about the role of ABC transporters in *P. aeruginosa* drug resistance [35], but it is clear that the presence of *C. albicans* alters the expression of several efflux pumps, which may contribute to carbapenem tolerance in dual-species biofilms. The *P. aeruginosa* genome encodes for at least 12 RND efflux pumps [36], 3 of which (MexAB-OprM, MexXY-OprM and MexCD-OprJ) have been linked to meropenem resistance [37, 38]. MexC was induced in dual-species biofilms in response to meropenem treatment, suggesting that *P. aeruginosa* may increase efflux through MexCD-OprJ. Phenazines and their derivatives have been shown to directly regulate the expression of several efflux pumps, including *mexG* [39, 40]. Therefore, increased synthesis of phenazines in these biofilms could alter drug efflux and increase the tolerance of *P. aeruginosa* to meropenem. In agreement with this, enhanced production of the phenazine, pyocyanin, promotes resistance to the beta-lactam class of antibiotics through reduced drug influx [41].

Other differences between the transcriptional profiles for single- and dual-species biofilms in response to meropenem include genes involved in biofilm formation (*pslH*, *pslI* and *algR*) and outer-membrane vesicles (OMVs). AlgR is a transcriptional regulator of genes involved in alginate production [42]. Therefore, in addition to enhanced extracellular polysaccharide production through Psl biosynthesis, dual-species biofilms will also contain increased levels of alginate. Increased alginate production is known to enhance biofilm production [22], but has not been directly linked to antibiotic resistance. OMVs play important roles in biofilms, including the secretion of PQS, extracellular DNA and beta-lactamase [43, 44]. Therefore, enhance OMV production in the presence of *C. albicans* may function to increase the composition and complexity of the extracellular matrix, which together with enhanced beta-lactamase secretion would increase the tolerance of *P. aeruginosa* to meropenem.

In summary, in response to meropenem, *P. aeruginosa* biofilms upregulate *ampC* and biofilm formation to provide protection from meropenem. Although dual-species biofilms exhibit enhanced tolerance to meropenem, the *P. aeruginosa* transcriptional responses from dual-species biofilms exposed to meropenem were similar to those of single-species biofilms treated with meropenem, suggesting that the presence of *C. albicans* in dual-species biofilms may limit the diffusion of or sequester the antibiotic, leading to increased bacterial tolerance (Figs 1–6).

## Supplementary Data

Supplementary material 1
