# Supplementary material for: Transcriptional profiling of Pseudomonas aeruginosa mature single- and dual-species biofilms in response to meropenem
Source: Microbiology (Reading). 2023 Jan 23;169(1):001271. doi: 10.1099/mic.0.001271 (PMC9993114; doi:10.1099/mic.0.001271)
Supplement: Supplementary material 1 [file mic-169-1271-s001.pdf]

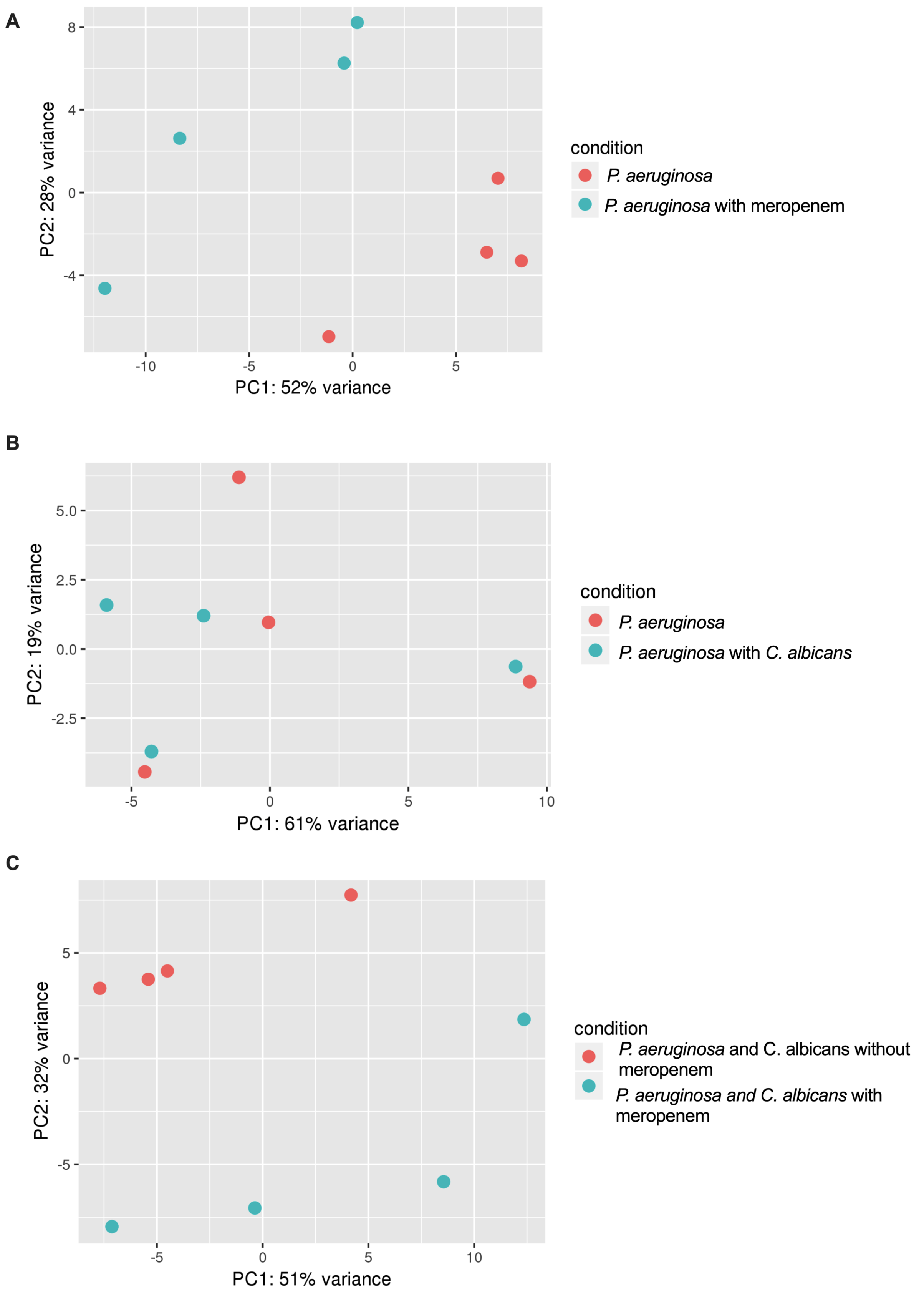

Supplemental Figure 1. PCA plots **A)** single species *P. aeruginosa* in response to meropenem. **B)** Dual species biofilms in the absence of meropenem. **C)** Dual species biofilms in the presence of meropenem.

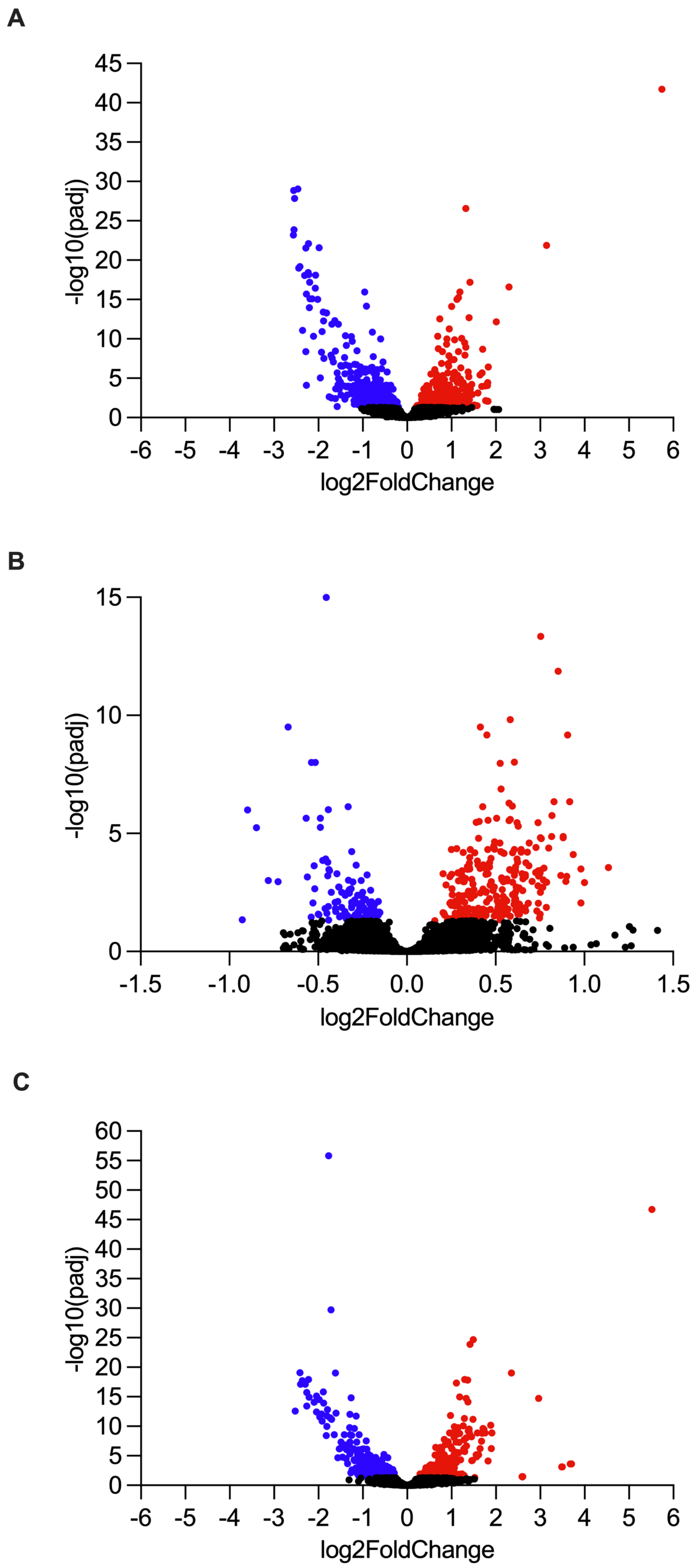

A
-log10(padj)
45
40
35
30
25
20
15
10
5
0
-6 -5 -4 -3 -2 -1 0 1 2 3 4 5 6
log2FoldChange
B
-log10(padj)
15
10
5
0
-1.5 -1.0 -0.5 0.0 0.5 1.0 1.5
log2FoldChange
C
-log10(padj)
60
55
50
45
40
35
30
25
20
15
10
5
0
-6 -5 -4 -3 -2 -1 0 1 2 3 4 5 6
log2FoldChange

Supplemental Figure 2. Transcriptional profiling identifies genes differential regulated in single and dual species biofilms in response to meropenem. Volcano plots identifying significantly differentially regulated genes in **A)** single species *P. aeruginosa* in response to meropenem. **B)** Dual species biofilms in the absence of meropenem. **C)** Dual species biofilms in the presence of meropenem.
